# Shape-shifting versions of class in Australia and the pursuit of equity in public health

**DOI:** 10.1093/heapro/daae093

**Published:** 2024-08-13

**Authors:** Megan Warin, Victoria Loblay

**Affiliations:** School of Social Sciences, University of Adelaide, North Terrace, Kaurna Country, Adelaide, South Australia 5005, Australia; Brain and Mind Centre, The University of Sydney, Gadigal Country, Camperdown, New South Wales 2050, Australia

**Keywords:** social class, health inequalities, anthropology, obesity, health promotion practice, social determinants of health, *habitus*

## Abstract

The COVID-19 pandemic and current cost of living crisis have highlighted socioeconomically patterned health disparities, bringing renewed focus on equity in public health. Despite political rhetoric invoking cultural narratives of egalitarianism and opportunities for class mobility, social class remains a significant factor in health outcomes in the Australian context. For social scientists, class (despite robust critiques) is a key analytical concept that has been theoretically broadened to encompass social and cultural practices (*habitus*). In public health, however, concepts of social disadvantage have expanded toward frames such as health equity and socioeconomic status in ways that can obscure ‘class’ and *habitus*. Understandings and operationalization of concepts of class and equity not only impact collaborative and interdisciplinary relationships, but also the framing of public health problems and health promotion interventions and policies. In this article, we draw on our experiences as anthropologists conducting ethnography in and of Australian health promotion programs to map and re-evaluate the intersection of concepts of social class and equity. We trace how representations of class emerged in these programs, and the versions of class and equity that materialized across different public health contexts. We argue for a conceptual repositioning of class that recognizes its shape-shifting qualities and of its materializations in different politics, disciplines and everyday contexts. In doing so, we highlight ‘class’ as a salient dimension of the design, implementation and evaluation of health promotion programs.

**Contribution to Health Promotion**

- Social class is a key concept that underpins health inequalities, yet is often overlooked in public health framings of equity and social disadvantage.
- Bourdieu’s *habitus* highlights how everyday social practices (e.g. eating habits, alcohol use, cycling) are linked to social class, challenging assumptions of many health promotion interventions.
- Appreciating different ways of understanding class enables deeper consideration of social disadvantage and the importance of critically interrogating social class *and habitus* when addressing equity in health promotion.
- Understanding the value of different approaches to social class and equity can inform health promotion program design, implementation and evaluation.

## INTRODUCTION

On 21 May 2022, a new Labor government under the Prime Ministership of Anthony Albanese took office in Australia. In opening his victory speech that night, Albanese emphasized where he had come from. ‘It says a lot about our great country’ he said, ‘that a son of a single mum who was a disability pensioner, who grew up in public housing down the road in Camperdown can stand before you tonight as Australia’s Prime Minister’ (*ABC News*, 2022). Albanese has often spoken to his working-class background and used his own biographical narrative of social mobility to put forward his political and social justice values, that ‘no matter where you live, who you worship, who you love or what your last name is, places no restrictions on your journey in life’. Like other Prime Ministers before him, his political identity is shaped by a keen awareness of the need to appeal to working-class people (Blaine, 2021). In closing his victory speech, he returned to his background, stating ‘I hope that there are families in public housing watching this tonight, because I want every parent to tell their child that no matter where you live or where you come from, in Australia the doors of opportunity are open to us all’. Bookended with values of fairness and opportunity, this narrative reflects a particular invocation of social class in the Australian context, one that taps into a cultural mythology of egalitarianism in the land of ‘the fair go’ (Blaine, 2021).

Social class is a concept that is difficult to define, with sociologist Wendy Bottero describing it as notoriously ‘slippery’ (Bottero, 2013). As an analytical category, it differs according to academic discipline, and changes across time (Savage *et al.*, 2013), politics and geographies. In appealing to a broad base, Albanese was drawing on a common-sense narrative that associates class with traditional classifications of occupation, location and income. UK sociologist of class Mike Savage suggests that the ways social class straddles both popular and academic discourse is what makes it distinctive: ‘The power of the class concept rests in its ambivalent location betwixt and between academic, political and public fields, and this is something to be celebrated as a means of recognising the fundamental political stakes tied up with the concept of class’ (Savage, 2016). As we detail in this article, social class narratives are contested and mired in a number of intersecting political discourses; the notion of social class can be pressed into service both through its elevation and its silencing. We position class as a shape-shifting category, acknowledging its intimate connection to social and health inequalities, and the ways in which it is politically mobilized in differing contexts and situations.

Whilst Albanese’s biographical story of class mobility and opportunity may be politically savvy, decades of public health research have produced alternative narratives through an ‘equity’ lens, whereby opportunities for flourishing and health are shaped by structural constraints and conditions of daily living, including food affordability and housing (Marmot, 2007; Commission on Social Determinants of Health, 2008; Baum *et al.*, 2009; Friel and Marmot, 2011). Albanese’s victory speech took place in the third year of the COVID-19 pandemic, when intersections of social and economic dimensions of virus transmission and disease burden had been made increasingly clear. Many migrants, and those in insecure, casualized jobs—which comprise largely of lower-paid jobs—were described by leaders as the ‘biggest driver’ of transmission rates during lockdowns (Teicher and Van Gramberg, 2020). At the same time that governments appeared to call attention to social determinants of health such as food security, work and housing, certain communities found themselves differentially targeted by stringent lockdown measures. Rapid changes to food supply, employment and household dynamics across the nation due to lockdowns impacted accessibility of foods, leading to higher prevalence of food insecurity for some groups (Foodbank, 2021). During the first year of the pandemic, there were almost four times as many deaths due to COVID-19 for people living in the lowest socioeconomic group compared with the highest socioeconomic group, and age-standardized mortality rates were 2.6 times as high (Australian Institute of Health and Welfare, 2021). These socioeconomically patterned disparities were noted in many other international contexts (Bambra *et al.*, 2020; EMG and NERVTAG, 2020; Suleman *et al.*, 2021), demonstrated in widening gaps in incomes, health equity, marginalization, food insecurity, employment and status. In areas of public health and healthcare, there has subsequently been a renewed focus on equity and social determinants of health (Brownson *et al.*, 2021).

These disparate depictions of health and social inequities on the one hand, and aspirational political discourses of egalitarianism on the other, reflect the contradictions and ambiguities that surround the conception of social class. Whilst neither directly deploy the language of ‘class’, we argue that they are nevertheless deeply mired in classed dimensions of inequality, widening wealth gaps and disadvantage. In their sociological analysis of public debates on class in Australia, Threadgold and Gerrard (Threadgold and Gerrard, 2022) observe that despite class operating as a ‘powerful political signifier’, in such political debates, ‘class is eclipsed; markedly absent’. This paradox of class has been highlighted by key scholars in both public health and sociology, who note that as economic inequality intensifies, popular awareness of class seems to wane (Savage, 2000; Savage *et al.*, 2013; Wacquant, 2013; Elliott *et al.*, 2016; Smith and Anderson, 2018). We trace the transformation of class in both social science and public health scholarship, and explore how ‘class decomposition’ (Tyler, 2015) has been shaped through a powerful politics that encourages individualization of social inequality.

**Fig. 1:**
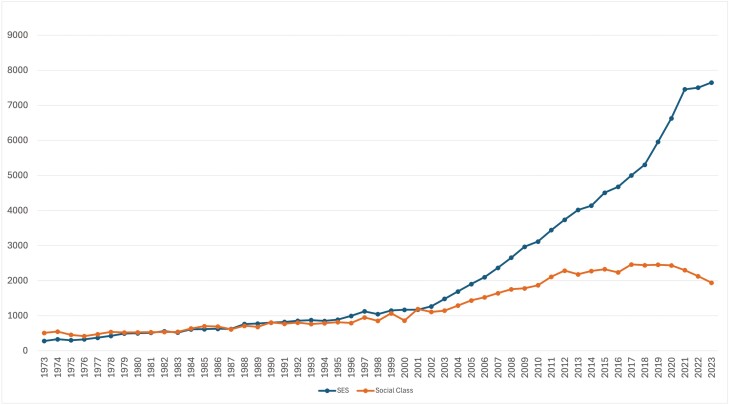
Scopus search (title, abstract, keyword) for ‘social class’ versus (‘socio-economic status’ OR ‘socioeconomic status’ OR ‘SES’) for medicine, health, social sciences, psychology and nursing 1973–2023.

How class is conceptualized has consequences for the ways in which class is researched, as well as the lived experiences of class. Findings from social science studies of class reveal how people experiencing disadvantage are unwilling to acknowledge the impacts of deprivation in efforts to resist the shame and stigma that accompany classism and poverty (Shildrick and MacDonald, 2013; Warin and Zivkovic, 2019). Researching class without reproducing this stigma thus presents a dilemma for researchers, and other descriptors that stand in for class, such as equity, have emerged. Instead of class as a descriptive category, researchers in public health fields describe the dynamics of health equity in terms of socioeconomic status (SES) indicators that reflect material conditions such as income, jobs and housing (Abel, 2007). The increasing trend toward research focused on SES rather than ‘social class’—illustrated in Figure 1—tends to foreground what is easily measurable at the expense of experiential, embodied, qualitative aspects of health inequalities (Elliott *et al.*, 2016). In documenting health disparities and measuring health equity, sociocultural factors are defined according to dimensions of identity or social group characteristics including ethnicity, race, gender, sexual orientation, religion or disability status (Braveman *et al.*, 2018; Hoyer *et al.*, 2022). Concepts of ‘social class’ may well be assumed, but the ways in which everyday lives are infused with class struggles and relations rarely figure as an explicit category for analysis (Muntaner, 2019). It is these everyday, shared social practices of class that we seek to highlight.

## ATTENDING TO SOCIAL CLASS ACROSS DISCIPLINARY FIELDS

As social anthropologists working in public health spaces in Australia, we recognize that the theoretical concepts that are familiar to our discipline (and underpin our approach to public health problems) are not necessarily shared or valued in all other disciplines. M.W. has two decades of experience working at the intersection of social class and obesity with a State and national health promotion intervention targeting obesity (Warin *et al*., 2008, 2015; Warin and Zivkovic, 2019; Warin 2023). This ethnographic work was undertaken in a South Australian location that experiences significant social and economic hardship, where social class pervaded everyday lives impacted by poverty, housing stress and food insecurity. V.L. has experience working at the health policy and practice interface, conducting ethnographic research with health policymakers, funders and health promotion practitioners in New South Wales (NSW) and Tasmania (Conte *et al.*, 2017; Loblay *et al.*, 2020, 2021a, 2022). Ethnographic work with health promotion teams at Local Health Districts across NSW showed how some teams prioritized equity in their implementation of a state-wide obesity prevention program, whereas other teams had different strategic foci such as relationship building or monitoring data (Grøn *et al.*, 2020). Further ethnographic research with community organizations and health promotion funders in Tasmania during the first COVID lockdowns explored how the crisis in food security was being leveraged to change the food system (Loblay *et al.*, 2021b). In these contexts, where people inhabited different roles and perspectives vis-à-vis the health problems being addressed and program implementation, notions of class emerged in multiple ways and in the guises of differing language.

We have observed ways in which the category of class has been leveraged, translated, transformed and (de)valued in health promotion practice and programs. On the one hand, we encountered resistance to our framing of public health problems in relation to social class, including how we might measure and monitor health promotion and health inequities, understanding everyday health ‘behaviours’ and practices, and the inclusion of social class in policy directives. On the other hand, we have noticed the language of SES and equity being invoked as an increasingly broad umbrella, with moral connotations that tend to deflect critical analysis. In our research, we uncovered how the flattening of social class sidestepped a series of important interconnected opportunities to understand and address equity; not only in the communities we worked with, but also in the knowledge making practices of the health promotion and policy workers, and dietitians and nutritionists who lead public health programs.

In the following, we provide important background to how the category of social class has been developed and critiqued in social sciences. We then turn to discuss how social class and public health work on class has intersected through the work of anthropologist Pierre Bourdieu, showing through our case studies how the category of class extends beyond social stratification to everyday cultural practices (Bourdieu’s *habitus*). Taking class as relational practice (and not a fixed category) reveals how class can encompass much more than social stratification; it is deeply concerned with social practices, struggles and resistance to classed imperatives. We highlight the gaps between this more expanded cultural version of class, offered through Bourdieu’s work, and the tensions that appear at the nexus of frontline health promotion practice, policymaking and interventions targeting disadvantaged communities. We explore the range of ways concepts of class and equity are invoked and consider instances where they retreat from view. We contend that social class—as a form of social practice, struggle and resistance—is often overlooked as frame of reference in many health promotion programs and policies. Subsumed under the language of equity and socioeconomic disadvantage, our conclusion considers what is lost when the lens of social class is obscured, and what can be gained when anthropological approaches to class are valued.

## WHERE HAS THE CONCEPT OF SOCIAL CLASS COME FROM (AND GONE)?

Class has a long history in sociological and anthropological theory and appears in many different disciplines (for example, social psychology, economics, political science, popular culture). Though we cannot do justice to this extensive literature here, it is important to note that there are conflicting schools of thought about class, differing theoretical underpinnings, and arguments about differing methodological approaches for measuring or ‘capturing’ such data. Rather than seeking to reconcile these multiple differences [sometimes referred to as ‘class wars’ (Savage, 2016; Threadgold and Gerrard, 2022)], we aim to provide a commentary on the range of social science approaches to class as a foundation from which to develop a productive conceptualization of class that can contribute to health promotion approaches to equity.

Many of the key contemporary resources that have examined social class in Australia come from Western scholarship and have done so within the disciplinary boundaries of sociology and anthropology. Broadly speaking, the three most prominent waves of academic thinking about class can be characterized as: (i) influenced by Marxist thought, (ii) focused on occupation as a defining feature and (iii) more recently broadened to cultural class analysis, including dimensions of social and cultural capital (Savage *et al.*, 2013; Sheppard and Biddle, 2017).

The roots of class as a category of analysis lie in 19th century sociology and the growth in industrial capitalism. The historical transformation brought about by capitalism was accompanied by significant theoretical thinking, most obviously by Marx and Engels, and in anthropology by Eric Wolf. Drawing on Marx’s treatise on economic production, Wolf (Wolf, 1982) argued that economic production and market relationships profoundly shaped the historical and unequal formation of power and class relations at local and global levels.

Social scientists in England were similarly writing on how the Industrial Revolution was increasing poverty and inequality. Booth’s detailed surveys on London’s neighbourhoods in the late 1880s entailed moral judgements about the poor being ‘drunken savages’, representations that continue to be associated with contemporary discourses of ‘welfare queens’ (Dow, 2015), ‘benefit scroungers … and white trash’ (Tyler, 2013). Occupation came to be seen as key determinant of class in Britain, and the linking of a man’s occupation to their class became a dominant mode of measuring and characterizing class in government and academic circles. British sociologist John Goldthorpe developed this occupational approach in the 1980s, and a refinement of occupational skills and wage conditions became the measurement tool in national (UK) and international surveys (the European socioeconomic classification) (Savage, 2016). Australian scholarship and popular discourse has largely followed these Global North waves in understanding social class.

Problems with this measurement of class emerged with significant changes to work, skills and gender relations across the 20th century, highlighting the limitations of linking class with the single axis of male employment. As women entered the workforce and the structure of households and families shifted, linking class to a man’s occupation became deeply problematic (Broom and Warin, 2011). In addition, fluctuations in the formal labour market led more people to be self-employed at home, or outside of such markets (for example, those who are retired, are carers or unemployed). Shifts in global wealth to the hands of relatively few no longer mapped onto occupational classes, with more recent scholarship suggesting that asset ownership, particularly of housing, is a stronger indicator of class than occupation (Adkins *et al.*, 2022).

In an Australian context, the intersections of social class and racism (both in terms of settler colonial histories and multiculturalism) were not always highlighted in academic critiques of the meaning and practice of class. As Behrendt, a Eualeyai/Kamilaroi academic describes, First Nations people were ostracized from the class structure through colonizing society and even ‘the myth of social mobility’ was out of reach:

You had to work to be white to have the same status as a working-class person, but really, you’d never get there because your race is always a barrier, and in country towns, Aboriginal people weren’t employed even when there were jobs. (Behrendt *et al.*, 2022)

In Australia, like many other liberal democracies, a ‘self-mythology about being a bastion of the fair go’ (Blaine, 2021) aligned with neoliberal narratives that diminished class as a determining social structure. Class, as noted by numerous social scientists, became a ‘zombie category’ (Beck and Beck-Gernsheim, 2002) and appeared to have been declared dead (Pakulski and Waters, 1997; Sheppard and Biddle, 2017; Threadgold and Gerrard, 2022) with widespread acceptance of the notion that anyone who chooses to work hard can climb the ladder. This discourse, as Harris aptly suggests, reflects an odd Australian dream that runs counter to sustained political inquiry—‘how could a settler colony founded on the destitution of its original inhabitants and the exploitation of imported convicts have ever been classless?’ (Harris, 2016).

This repositioning of class needs to be situated within major shifts in global politics, which underscores how politicians approached the issue of health inequalities. Thatcher, for example, attempted to bury the 1980 Black Report as she was displeased by the key finding that inequalities in Britain were due to class and income disparities (Black *et al.*, 1980; Commission on Social Determinants of Health, 2008; Lynch, 2020; Marmot, 2022). Downplaying the report by referring to inequalities as ‘variations’, the Thatcher government ‘denied that health inequalities were caused by material factors and attributed them to statistical artifact, social mobility, or, more often than not, individual behaviour’ (Baggott, 2010; cited in Lynch, 2020). It was during this political climate of a transition from welfare to neoliberal economies and reforms in the 80s and 90s (under Thatcher *and* New Labour) that the neoliberal paradigm of Europe [and Australia] came to prominence (Lynch, 2020). Despite attempts to keep health inequalities on the agenda, values of individualism, choice, free markets and competition place an emphasis on personal responsibility and lifestyles (Baum and Fisher, 2014) continuously challenging the design of health promotion agendas focused on creating supportive environments and strengthening community actions (Fry and Zask, 2016).

## MAPPING INTERSECTIONS OF SOCIAL CLASS AND PUBLIC HEALTH

In public health, particularly within epidemiology, measurement of social class became enmeshed with measurements of socioeconomic inequality as part of efforts to explain observations of class differences in morbidity and mortality (Najman, 1988). However, a lack of consensus on definitions of social class and SES meant that operationalizing these notions through measurement was rarely based on theory (Oakes and Rossi, 2003). With low correlations between single measures of class, such as ranking people by occupation, and prediction of health status, a range of class indicators including occupational skill, status, subjective assessments and income were devised (Abramson *et al.*, 1982; Najman, 1988). At the same time, these SES measures were not able to properly capture ‘the richness of the embedded social context’ crucial for the broader field of public health (Oakes and Rossi, 2003). Newer analyses of class were needed for shifting gradients of class in a post-industrial societies, in order to understand how class inequalities were generated in *every sphere of social life*.

A broader understanding of social class came to the fore in the 1990s with the translation of anthropologist Pierre Bourdieu’s work on French society. In his classic treatise on French social class entitled *Distinction: A Social Critique of the Judgement of Taste*, Bourdieu (Bourdieu, 1984) challenged social stratification theory by pointing to the limits of placing objective systems of measurement over people. He rallied against preordained classifications of class that rendered groups of people passive, instead focusing on how social classes only emerge through social practices and struggles—and are always relational.

As well as the more traditional forms of struggles between labour and capital (as in Marxist analyses of class), Bourdieu showed how class struggles are also cultural, arising in everyday and mundane practices of tastes and capital. He identified different forms of capital—economic, cultural and social. These differing types of capital have differing values. They come together to form a *habitus*, a structure in which patterns of consumption in all manner of tastes (e.g. from home decor, fashion, the books we read, the schools we attend, choice of car and parenting styles) are unconsciously embodied to orient us in the world, structuring everyday lives (Bourdieu, 1977; Warin *et al.*, 2008; Bennett *et al.*, 2020). These three capitals may not be evenly distributed, but can be leveraged to demonstrate performances of gender, power and identity, and in doing so create hierarchies of social class, distinction and privilege (Bourdieu, 1984) which are used ‘as weapons and prizes in the struggle between the classes’ (Skeggs, 2013).

By the late 1990s, health promotion began to recognize the potential of Bourdieu’s social capital theory for addressing the links between social inequalities and health (Hawe and Shiell, 2000). However, despite the enthusiasm for social capital research, qualitative differences between macro/context level and micro/individual level remained under-appreciated (Hawe and Shiell, 2000). As such, challenges facing epidemiological measurement of class and appreciation of social contexts were not necessarily resolved through the application of social capital theory. Here, we see the possibility for a renewed focus on Bourdieu’s contribution to social class and health by drawing in the concept of *habitus* and social/cultural capital to both health promotion interventions and the communities in question.

Food, drinking, exercise and health are very much a part of one’s *habitus* and are strongly influenced by differing social and economic capital (Bourdieu, 1984; Warde, 2016). A recent study examining disparities in French children’s food tastes clearly demonstrates how *habitus* profoundly shapes food and eating practices (Lebredonchel and Fardet, 2023). Children from higher socioeconomic classes displayed a *habitus* that was influenced by links between food and ‘good’ health and were more likely to discuss foods that were ‘organic’, ‘homemade’ of ‘free from chemicals’. Children from lower socioeconomic classes rarely discussed links between food and health and had more limited food repertoires than their counterparts. These differing *habituses* and forms of cultural capital were contextualized by classed parental styles (as well as ethnicity), such as desires to expose children to different food experiences (through travel or expanded cuisine), or in the case of the lower-class families, to simply provide foods that were filling and pleasurable, and shielded against deprivation.

Bourdieu’s *habitus* and cultural capital framework has been valuable for thinking about other interconnected classed health practices, for example healthy transport and mobility practices (Steinbach *et al.*, 2011; Nettleton and Green, 2014), obesity (Warin and Zivkovic, 2019) and alcohol use (Lunnay *et al.*, 2022); all highlighting how everyday consumption practices are profoundly shaped by one’s gendered, racialized and classed *habitus*.

In recent ethnographic work exploring Australia’s largest childhood obesity intervention, Warin and Zivkovic found that ‘healthy foods’ promoted by middle-class health promotion workers were completely at odds with the *habitus* of community participants who had limited resources and shopped at food banks (Warin and Zivkovic, 2019). In a context where financial constraints determined food choices and food insecurity was high, recommendations to eat expensive summer fruits such as blueberries or strawberries were out of reach—and finances trumped nutritional priorities. When ingredients like coriander were suggested to add to recipes, local families exclaimed that this was a taste (both sensually and aesthetically) outside of their familiar *habitus*, and something they ascribed to ‘toffy’ or ‘more refined’ people. Aspirations for bodily thinness were also remarked upon by women in this study, as only people who were sick were thin, and extra body fat was described as a buffer when food was scarce (Zivkovic *et al.*, 2018). Class, like gender, is embodied through a socially and spatially located *habitus*, and becomes a marker and performance of taste and social/cultural capital. Importantly, the middle-class *habitus* that underpinned views of ‘healthy eating’ held by the health promotion workers was taken-for-granted and normalized in this intervention.

The resistance to healthy eating clearly communicates class values. While the word ‘class’ might not itself arise in that context, these examples demonstrate how health inequalities were presented as classed struggles. The resistance to expensive summer fruits, the assumptions that people in the community needed to be taught how to ‘eat right’ or the judgements about the amount of sugar and salt in food were all struggles over classed values attributed to differing *habituses*. As Imogen Tyler (drawing on Bourdieu) suggests in her work on class in the UK, ‘class is an operator of conflict’ (Tyler, 2015). And these refusals of middle-class admonishments were claimed as de-stigmatization strategies by participants in M.W.’s research, pushing back against class stigma.

Class identities in M.W.’s research were often presented and performed with pride. The weathering of intergenerational hardships was key to countering the negative representations that outsiders often attributed to these ‘working class’ and ‘welfare’ neighbourhoods. Many families described a pride in knowing how to ‘live poor’, of being able to source the cheapest foods available, selling and giving away long-life foods and locally grown fruits and vegetables from garages and verandas, living to the cost-saving strategies of the Great Depression, and working around the high prices of gas and electricity. For one family that had experienced a period of homelessness with five children, the joy of now being able to fill their shopping trolley, fill their car boot and then fill their cupboards at home demonstrated their capacity as parents to be able to feed and care for their family. Overflowing freezers and cupboards, stuffed with ready-made pizzas and bottles of soft drink were welcome material measures of how far this family had come from empty shelves and cupboards (Warin *et al.*, 2019).

Children in this research similarly recounted pride in resisting public health imperatives that encouraged them to ‘choose healthy lifestyles’ and ride their bikes to school. This community was built on the back of Australia’s national car manufacturers (Holden), employing generations of families before their closure in 2017. This history was embodied as a classed *habitus*, with one child stating that only ‘kids who are povo [poor]’ ride their bikes to school. For these children, riding a bike signals poverty, whereas arriving at school in a car symbolizes status, working class pride and cultural capital. In recounting findings of social class back to the program managers of an obesity intervention, MW encountered a reluctance to accept that class was an important factor in people’s experiences. She discussed the middle-class imperatives of lifestyle change messaging around food and obesity, and the constraints on people’s ability to choose ‘healthy foods’ when their priority was simply to deal with hunger. Despite the fact that this public health obesity intervention had targeted a community designated as ‘disadvantaged’, and the significant impact of poverty, hunger and hardship on everyday lives, M.W. was asked by managers of the health promotion program, ‘What’s class got to do with it?’

## TRANSLATING CONCEPTS OF CLASS AND EQUITY IN PUBLIC HEALTH

Though there may be reluctance to engage directly with concepts of social class in some facets of public health, the language of health equity, social determinants of health and SES are much more familiar. There have sometimes been calls for increasing clarification of these terms, for example, distinguishing equity from inequality (Brownson *et al.*, 2021) or health differences from health disparities (Braveman, 2014), but the validity of these terms as relevant for public health priorities is rarely questioned. Yet these definitions tend to be couched within the frame of social and/or economic disadvantage:

Social disadvantage is a broader concept. While it includes economic disadvantage, it also refers more generally to someone’s relative position in a social pecking order—an order in which individuals or groups can be stratified by their economic resources, as well as by race, ethnicity, religion, gender, sexual orientation, and disability. (Braveman, 2014)

The classed concept of *habitus* and associated struggles over differing forms of capital, is notably absent in this articulation of ‘a social pecking order’.

For public health practitioners working at the frontline of interventions, however, concepts of class can often erupt as points of tension in pursuit of equity goals. This is particularly true when interventions are designed to reach a target population classified as disadvantaged. In 2017, V.L. was part of a team doing ethnographic fieldwork describing the scale-up of a $45 million obesity prevention program in schools and childcare centres in NSW. For the first time ever, health promotion practitioners had to report to state-level policymakers on program implementation and meet ‘key performance indicator’ (KPI) targets. In spending time with practitioners who were tasked with delivering the health promotion programs, driving with them in cars, attending school workshops and watching them fill out monitoring data, issues of ‘equity’ and ‘class’ hovered frequently in conversations. In some teams, equity appeared as a central driver of decision-making, using resources to assist schools in areas classified as ‘low SES’ (a measure of SES) (Grøn *et al.*, 2020). We heard that KPI targets sometimes made it more difficult for practitioners to prioritize schools which were struggling or disadvantaged. Some practitioners described the programs as very ‘Middle Class’. Devising health education materials (bespoke pamphlets for example) for different communities was one way that practitioners tried to fine-tune the state-wide programs to meet local needs (Loblay *et al.*, 2020).

At the policy level, a different picture of equity emerged. Program managers used monitoring data to measure the ‘equitable reach’ of the programs. Using targets (set at 80%), they deemed that equitable reach was achieved for schools in areas of socioeconomic disadvantage, schools in remote areas and schools with a high proportion of Aboriginal students (Bravo *et al.*, 2020). So, on the one hand, practitioners were describing the challenges of making state-wide programs ‘fit’ with equity agendas and professing concerns that the programs were ‘Middle Class’. On the other hand, program managers were using monitoring data to claim that equitable reach was accomplished because enough schools with the hardest to reach populations were achieving the target proportion of program practices.

In some ways, such divergent interpretations of equity are to be expected within the field of public health. They reflect different priorities of policy-based practice compared with practitioners tasked with implementing interventions. It is also possible, however, that this flexible use of equity serves as a shroud that obscures differences in experience that ought to be confronted. Would an analysis of equity that integrated a more developed conceptualization of social class allow for a more productive discussion around the intersection of equity and social disadvantage?

## CONCLUSION: REIMAGINING WAYS OF WORKING WITH ‘CLASS’ IN PUBLIC HEALTH

The field of public health has made significant policy gains (education, taxation etc.) through highlighting the health gradient associated with social determinants of health. Yet while measurement-based manifestations of social disadvantage (such as SES) may be helpful for depicting different health trajectories for different groups, they are less helpful for design and implementation of on-the-ground programs which engage with people in communities. The lens of our ethnographic experiences affords us a view of how equity is readily adopted and espoused as a way to demonstrate intervention efficacy, while concepts of social class persist in particular contexts, even as they are simultaneously hidden from view. In this sense, class and equity are concepts that have practical enactments. They are embodied, discursively produced, move, offend and speak to power. It is important to hold these differences of how class and equity materialize in different sites, be they offices of State health departments, the kitchens of participants or the words of policy documents.

We contend that reinstating Bourdieu’s concept of ‘class’ as a culturally practiced, analytical category in relation to health equity means embracing the *capacity* of the concept rather than pursuing its specificity. Instead of relying on social class as a fixed category, we suggest an approach that allows for the multiple ways the concept is enacted as it moves across contexts. This is what Mol and Law (Mol and Law, 1994) have termed ‘a mutable mobile’ or a ‘fluid object’ which changes over time, along with its component parts. As class is practiced in different situations, the concept itself becomes different and may take on different meanings. As a marker of cultural identity and pride, class is not defined by health indicators but may be distinguished by care for family and thrift. But when a working-class community becomes entangled in an obesity prevention program, class is designated by SES or disadvantage. A health promotion practitioner narrating the story of program implementation may frame class as a barrier to program implementation. In these ways, reimagining class does not entail translation of one concept, but allowing for differing versions and appreciating how these versions emerge in different sites and the tensions between them.

Public health scholarship on health equity emphasizes fairness and justice as defining characteristics of equity (Whitehead, 1991). If these values are to be realized in tackling health disparities in pursuit of equity through public health interventions, we need new ways of working in communities where social class features as a salient feature of context. SES or ‘disadvantage’ may be insufficient for capturing the multiple versions of social class in intervention contexts. As health promotion policy-making increasingly incorporates community consultation and co-design, acknowledging the broader ‘social currencies’ of *habitus* of communities and our own taken-for-granted *habitus* on what constitutes ‘good health’ is more pressing than ever. Ethnographic methods, alongside co-design and collaborating with communities, offer possibilities for capturing community-based meanings of social class that account for cultural tastes and struggles. This offers a possible first step in rethinking approaches to public health interventions in ways that allow cultural analyses of class to be redefined as a potential community resource, rather than simply a barrier or disadvantage.
